# Reliable Link Level Routing Algorithm in Pipeline Monitoring Using Implicit Acknowledgements

**DOI:** 10.3390/s21030968

**Published:** 2021-02-01

**Authors:** Carlos Egas Acosta., Felipe Gil-Castiñeira, Enrique Costa-Montenegro, Jorge Sá Silva

**Affiliations:** 1 GI-IoE Internet of Every Things Research Group, Escuela Politécnica Nacional, Quito E11-253, Ecuador; carlos.egas@epn.edu.ec; 2 atlanTTic Research Center for Telecommunication Technologies, Universidade de Vigo, Information Technologies Group, 36310 Vigo, Spain; kike@gti.uvigo.es; 3 Department of Electrical and Computer Engineering, INESC Coimbra, University of Coimbra, P-3030 Coimbra, Portugal; sasilva@deec.uc.pt

**Keywords:** access protocols, industrial communication, wireless sensor networks, linear networks

## Abstract

End-to-end reliability for Wireless Sensor Network communications is usually provided by upper stack layers. Furthermore, most of the studies have been related to star, mesh, and tree topologies. However, they rarely consider the requirements of the multi-hop linear wireless sensor networks, with thousands of nodes, which are universally used for monitoring applications. Therefore, they are characterized by long delays and high energy consumption. In this paper, we propose an energy efficient link level routing algorithm that provides end-to-end reliability into multi-hop wireless sensor networks with a linear structure. The algorithm uses implicit acknowledgement to provide reliability and connectivity with energy efficiency, low latency, and fault tolerance in linear wireless sensor networks. The proposal is validated through tests with real hardware. The energy consumption and the delay are also mathematically modeled and analyzed. The test results show that our algorithm decreases the energy consumption and minimizes the delays when compared with other proposals that also apply the explicit knowledge technique and routing protocols with explicit confirmations, maintaining the same characteristics in terms of reliability and connectivity.

## 1. Introduction

Many industrial applications require the deployment of sensor networks that follow straight lines. For example, in applications such as monitoring water, oil, or gas pipelines, roads, tunnels, borders, etc., wireless communication technologies provide clear advantages (such as self-organization, rapid deployment, cost, or flexibility) for the creation of highly reliable and self-healing industrial systems that can respond to new events [1]. This problem has been studied in recent years, and different authors proposed the deployment of Linear Wireless Sensor Networks (LWSN) [2], characterized by sparse node deployment along the linear infrastructure. It is assumed that nodes cannot move after being deployed. One of the main constraints in linear topology is the reduced number of neighbor nodes that limits the feasible transmission routes [2]. Such limitations make traditional solutions for Wireless Sensor Networks (WSNs) inapplicable to LWSNs [3]. For example, complex routing protocols may adversely affect the performance of the sensor network [4], and the limited number of transmission routes may increase the data loss probability [5].

Nevertheless, there are still important challenges that must be addressed. For example, in order to avoid changing batteries (which may be a complex or expensive procedure), power consumption must be optimized [6]. Such infrastructures may cover hundreds or thousands of kilometers, so it is necessary to consider end-to-end delay, node placement, and routing.

Different proposals have been developed to optimize the power consumption of the nodes and minimize delays. Many studies have focused on minimizing energy consumption during transmission and reception [7], while others have focused on minimizing the processing of information in the sensor node [8], a task which can also increase the delay.

In LWSNs, nodes may be placed in a thin (one dimension, forming a line) or thick (two or three dimensions around a line) configuration, and can have identical (one-level) or differentiated (multiple-level, creating a hierarchical network) functionalities [9]. In the applications such as pipeline monitoring, a thin one-level network is cheaper and simpler to build and maintain, this being the configuration considered in this paper to propose solutions for the minimization of energy consumption and delays (while maintaining reliability and connectivity) in LWSNs. Nevertheless, a direct transposition of classical protocols on LWSN is not efficient, as linear topologies are the worst cases for hierarchical networks (spoilage) and for stochastic distribution (time for reparation) [10]. Hence, routing protocols designed for nonlinear WSNs should not be directly applied to LWSNs with hundreds or thousands of nodes due to their special topology and application requirements [11]. For example, IEEE 802.15.4 nodes can use ACKs to confirm the reception of a frame. This technique is known as explicit confirmation (eACK), but the transmitting node can also receive an implicit confirmation (or iACK) when it receives the same information after the receiving node retransmit it to the next hop. 

By using implicit acknowledgements (iACKs), we can address two challenges: reducing energy consumption and latency. This technique helps to decrease the packet exchange between nodes, and to reduce the communication stack by removing most of the functions usually implemented at network level layer (OSI layer 3). This is possible because in a wireless network when a node transmits a frame, all the active nodes in the coverage area receive it and in addition, in a linear topology, the routing is very simple, as packets can only go in one of the two directions of the linear infrastructure, and nodes just have to decide if they have to retransmit a packet or not. Thus, networking functions that are typically implemented in a separated layer, can now be integrated within layer 2 (link) functionalities. The proposed algorithm for LWSN was validated through real-world testbeds and contributes to the following aspects:

- Reliability of the transmission, using iACK.
- Reduction of the delay time caused by using acknowledgement frames.
- Alternative nodes are selected to transmit when failed nodes or links occurs, without using routing.
- Reduction of computation in the node thanks to the elimination of routing tasks.
- Automatic assignation of addresses to nodes using link level processes.
- Reduction of the energy consumption during the end-to-end reliability transmission process.
- Improvement of the scalability with a large number of sensor nodes in LWSN, allowing for the insertion of new nodes automatically.
- Maximization of the entire system’s lifetime due to the energy efficiency in each node.

The paper is organized as follows: Section 2 discusses the related research; Section 3 presents the proposed algorithm; Section 4 analyzes the energy and the transmission time; and Section 5 describes a real implementation. We conclude our paper in Section 6.

## 2. Related Work

WSNs have been widely studied, and there are different protocols that have been designed to support a wide range of network topologies (star, mesh, etc.), for example, 6LowPAN and ZigBee, which can use IEEE 802.15.4 for the lower layers. Nevertheless, linear networks have very specific requirements which are not well addressed by generic protocols, as shown in [12], where the authors review and compare different aspects of routing protocols (addressing policies, discovery mechanisms, routing, etc.) designed specifically for LWSNs or for more traditional WSNs, by analyzing metrics such as end-to-end delay, reliability, or deployment requirements. Therefore, we can conclude that LWSNs require specifically designed communication stacks. In this sense, there are different proposals for new MAC layer protocols for LWSN. LC-MAC [13] is a long-chain MAC protocol where the forwarding nodes schedule the transmission of packets to reduce the delay of an end-to-end extended sensor network without sacrificing energy efficiency. CH-MAC, a delay-aware MAC protocol with implicit control frame Request to Send (RTS) employed in the medium access control, is proposed and compared with eACK in [14]. The authors conclude that it prevents the “avalanche” effect, and the use of iACK optimizes the use of energy. In [15] a token-based MAC protocol for linear sensor networks has been proposed to manage the access to the medium and improve the network performance. Also, many protocols have been presented to optimize the energy consumption of large WSNs. For example, in [16] MI-MAC, a new link level protocol, is proposed. Finally, reference [17] includes an analysis and comparison of different MAC protocols for linear LWSN such as Dual mode MAC, Disc-MAC, LC-MAC, MFT_MAC, CMAC-T, AC_MAC/DPM, and WiWi protocols.

Different routing protocols have also been suggested to meet the specific LWSN needs. In [10] a robust discovery, addressing and routing protocol for dynamic LWSN is proposed. The application of LWSN to rail transport is considered in [18], and energy efficient routing protocols for linear wireless sensor networks are also studied. The authors recommend two energy efficient routing protocols, Minimum Energy Relay Routing (MERR) and Adaptive MERR (AMERR). A Chain-based Anonymous Routing (CAR) unicast-based broadcast to send data is studied in [19]. In this proposal, each node can only sense the adjacent nodes on the same chain, and the traceability of the system has been enhanced; thereby increasing network security. In [20] a new routing algorithm is proposed for wireless sensor networks for post-disaster road monitoring. For this approach, the nodes are arranged in a linear topology. It includes route establishment, packet forwarding, and route maintenance. This proposal assumes that the nodes are previously identified in sequence. Finally, the AODV Routing Algorithm in Linear Topological Wireless Sensor is studied in [21].

Transport and application protocols have been also developed for WSNs to guarantee reliability, flow control, and congestion control. However, large-scale networks require a complex implementation complicated by the limited processing and power resources of the nodes. For pipeline monitoring the main issue is reliability, since controlling the monitoring period helps to minimize other problems such as congestion or flow control. For example, a comparative analysis of the transport and application protocols CODA, ESTR, RMST, PSFQ, GARUDA, and ATP, proposed for large-scale WSN networks, is presented in [22]. The comparison includes the use of end-to-end ACK and NACK, but the studied protocols do not use eACK or iACK at the link level. The Rate-Controlled Reliable Transport (RCRT) protocol is presented in [23]. It uses a base station or receiver to discover missing packets and explicitly requests the packets from the sensor nodes for end-to-end recovery. The Stream Control Transmission Protocol-WAN (SCTP-WSN) [24] adopts the concept of Multihoming and adopts the SCTP transport protocol combined with the AODV routing protocol to provide network reliability with eACK. GARUDA [25] studies the reliability of the delivery of point-to-multipoint data in the downstream direction, from a sink to the sensor nodes using NACK. The Aggregation and Transmission Protocol (ATP) transport protocol [26] includes an end-to-end feedback control algorithm assisted by the receiving node and the network to counteract packet loss, using selective ACK. 

Nevertheless, using end-to-end mechanisms for reliability has also drawbacks, such as a higher delay because of the end-to end retransmissions. In [27], the authors study the advantage of using hop-to-hop, instead of end-to-end acknowledgment, and in [28,29] the authors study the advantage of using iACK, instead of eACK in wireless sensor networks. Based on these conclusions, our proposal decreases the energy consumption and minimizes the delays using iACK for the communication. It provides the same reliability, end-to-end connectivity, and scalability as previous proposals that implement high level routing protocols and eACK mechanisms.

We take advantage of the previous work and we propose a new cross-layer algorithm for LWSNs, which unifies MAC, routing, and address assignation. This algorithm also provides end-to-end reliability and solves the hidden nodes problem without requiring ACK, RTS, or CTS signaling. Nevertheless, our proposal combines several advantages compared with previous proposals, such as the presented in [12], where the authors review different LPWAN protocols considering static and mobile nodes. 

Table 1 compares our proposal with the protocols presented for static nodes and some other proposals specifically designed for large scale linear infrastructures. It is important to note that all the studied protocols at the link level use eACK, while our proposal uses iACKs because of the benefits provided for linear infrastructures. 

Thus, our work is essential to facilitate the deployment of simple and cheap LWSNs, pave the way for the implementation of new services and applications [30] and take advantage of new paradigms such as fog computing [31], cloud computing [32], and the analysis of large amounts of data [33] also in LWSNs. 

## 3. Proposed Algorithm

Our algorithm is specifically designed to address the capture of data and its transmission in linear infrastructures. Although many infrastructures are not strictly linear (e.g., a pipeline or a road may have branches), they can be separated in linear segments. Therefore, we make the following assumptions:

- We consider a linear structure, without branches, with *n* nodes and two border nodes or base stations, *v_0_* and *v_n+_*_1_, which are responsible for sending information to the central monitoring using long-range communication protocols.
- Each node works with the IEEE 802.15.4 standard in beaconless mode (CSMA/CA).
- Each node has a coverage area of 50 m (a reasonable distance for an IEEE 802.15.4 network in the 2.4 GHz band [43]), without obstacles and transmission conditions in the line of sight, and is associated with four nodes, two on the left and two on the right.
- The nodes are deployed with a separation of 25 m. Therefore, a node has at least 4 nodes in range.
- The participating nodes use a predefined PAN identifier to prevent the connection to other nodes that are not part of the linear infrastructure.
- Each node has an identifier assigned sequentially and automatically using processes at the link level, as indicated in [44].
- The bits of the header identified for future use are used to implement the control messages required by the proposed algorithm.

**Figure 1:**
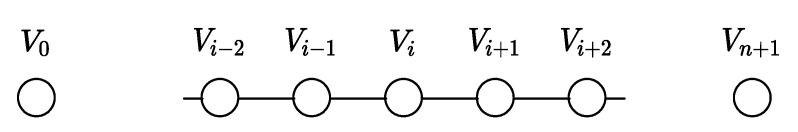
Linear Wireless Sensor Networks.

We propose using LWSN topologies as the one shown in Figure 1 which consists of *n* + 2 nodes, sequentially identified from 0 to *n* + 1. The sequential assignment of the identifiers is an essential feature on this research approach and solved as detailed in [44]. 

Nodes *v*_0_ and *v*_*n*+1_ are connected to the control center and do not have any limitation on energy or communication technologies, while nodes *v*_1_ to *v_n_* are nodes with sensors that capture information and also retransmit the frames of the neighboring nodes. Sensor nodes generate traffic when an event happens (e.g., the rupture of oil pipelines, unauthorized entry into borders, etc.). The information generated by node *v_i_* is transmitted to the neighboring nodes, creating two traffic streams simultaneously, *P_l_* (left direction) and *P_r_* (right direction), so it can reach the control center through *v_o_* and *v_n+_*_1_. This feature can be used to introduce redundancy in the network, but the operation with both flows is similar, so we are going to study only the *P_r_* flow. The nodes are in fixed locations, and therefore, the topology does not change in time. Modifications to the topology will only be made when a node or several nodes fail, or a link failure occurs. This feature allows the use of simpler routing algorithms and facilitates the location of nodes.

### 3.1. Algorithm Implementation

Each node includes four buffers (*buffer_tx_*, *buffer_rx_*, *buffer_ok_*, and *buffer_adj_*) and will always check if the received frame is stored in one of them to perform the appropriate actions. *Buffer_ok_* stores the transmitted frames that have successfully arrived at the destination node; *buffer_tx_* stores the transmitted frames from which a successful transmission has not been confirmed; *buffer_rx_* stores the frames received; and *buffer_adj_* stores the frames that have been sent by an adjacent node. The node *v_j_* is adjacent to node *v_i_*, when |*j* − *i*| ≤ 2. 

**Figure 2:**
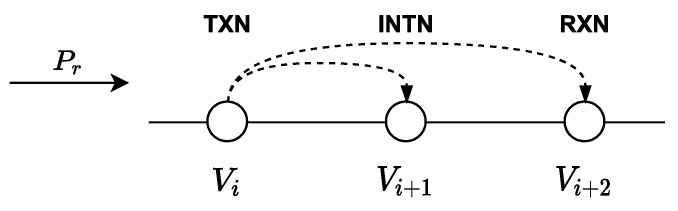
Node identification.

As shown in Figure 2, the transmitting node *v_i_* is called TXN, node *v*_*i*+1_ is called INTN (intermediate node) and node *v*_*i*+2_ is called RXN (reception node). When a TXN node transmits a frame, a *timer_tx_* is started. When an INTN node receives a frame, it starts a *timer_int_* whose minimum value is equal to the time necessary for the RXN node to receive and retransmit the frame. When the node *v_i_* detects an event, it sends information to the control center, acting as a TXN node, transmitting a broadcast frame. When node *v_i+_*_2_ receives a broadcast frame with flow *P_r_* (towards *v_n+_*_1_) and if source address is *v_i_*, the node *v_i+_*_2_ it will work as a RXN node. The frame will include the variable *FailN* that will have been activated if the node *v_i+_*_2_ fails, and the variable *ChangeF* that indicates a change in the data flow; both variables are initialized with “FALSE”.

**Figure 3:**
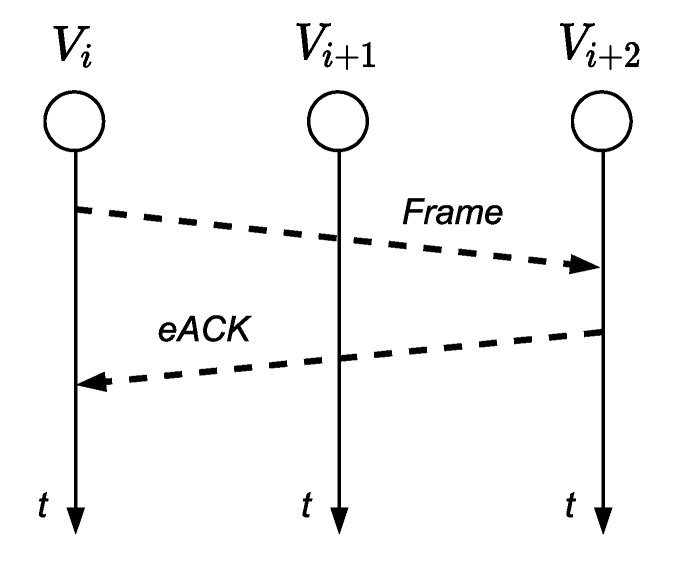
eACK.

There are two options to acknowledge the reception of the frame by the RXN node: using explicit ACKs (eACK) or implicit ACKs (iACK). In the first case, when the RXN node successfully receives a frame from TXN, it responds with an ACK, as shown in Figure 3. This approach increases the usage of energy (to explicitly transmit the ACK) and may even increase the latency if we wait for the ACK before transmitting a new frame. In 802.15.4, the additional time that node *v_i_* must wait to transmit another frame when an ACK is used is defined by the time to pass from transmission mode to reception (TTA), the duration time of the ACK frame (which involves the additional back off time in the node *v_i_* associated with the retransmission of the frame to the *v_i+_*_2_ node), and the time for the Clear Channel Assessment (CCA).

**Figure 4:**
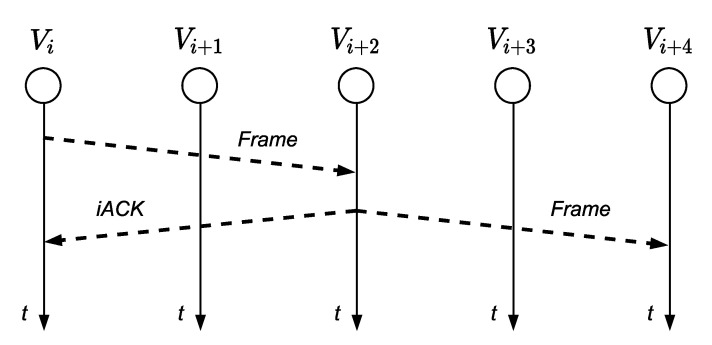
iACK.

Nevertheless, when the RXN node sends again the frame towards the destination (*v_n+_*_1_), the nodes in the surrounding (*v_i_*, *v_i+_*_1_, *v_i+_*_3_ and *v_i+_*_4_) will receive the information. Therefore, TXN will know that its frame has arrived at node *v_i+_*_2_ because it has retransmitted it. This works as an implicit acknowledgment (iACK) of the frame sent by node *v_i_* and avoids the need for the transmission of an ACK from *v_i+_*_2_ (RXN), as shown in Figure 4.

**Figure 5:**
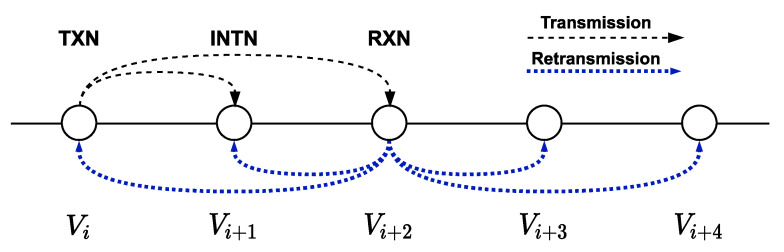
Node *v_i_* transmits to node *v_i+_*_2_.

In order to summarize, Figure 5 presents a typical scenario. When node *v_i_* detects an event, its objective is to transmit this information to the central station, so it transmits the broadcast frame to the node *v_i+_*_2_, and the node *v_i+_*_1_ also receives the frame. The node *v_i_* stores the frame in *buffer_tx_*. Nodes *v_i+_*_1_ and *v_i+_*_2_ store the received frame in *buffer_rx_* and *v_i+_*_2_ also verifies if it comes from an adjacent node to store it in *buffer_adj_*.

The frame retransmission from node *v_i+_*_2_ to node *v_i+_*_4_ is also received by nodes *v_i_*, *v_i+_*_1_ and *v_i+_*_3_. When *v_i_* receives the retransmission from node *v_i+_*_2_ to node *v_i+_*_4_, it checks if the frame is stored in one of its buffers. If it is stored in *buffer_tx_*, the node concludes that its transmission was successful without the need of an ACK from *v_i+_*_2_, so it stores the frame in *buffer_ok_* and eliminates the *buffer_tx_* frame. If the frame was stored in *buffer_ok_*, it is discarded because it is confirmed that the frame was received by *v_i+_*_2_. When node *v_i+_*_1_ receives this frame, it verifies if the frame is already stored in any of the buffers. If the received frame is stored in *buffer_rx_*, node v*_i+_*_1_ stores the frame in *buffer_ok_* and removes it from buffer_rx_. If it is stored in the *buffer_ok_*, it discards it because it is confirmed that the frame was received by *v_i+_*_2_. Since this frame was not transmitted or retransmitted by the *v_i+_*_1_, it will not be stored in the *buffer_tx_*.

### 3.2. Algorithm Robustness

A noisy link prevents the signal from being picked up by the receiving node and therefore the frame cannot be recovered and retransmitted to the next node. In 802.15.4 the unslotted mechanism may cause collisions. However, the unsynchronized behavior of the back off mechanism reduces the possibility of simultaneous transmissions, causing the Rx-to-Tx turnaround to be the main reason behind the collisions. The non-negligible Rx-to-Tx turnaround time in the unslotted mechanism may cause collisions between data frames [45].

**Figure 6:**
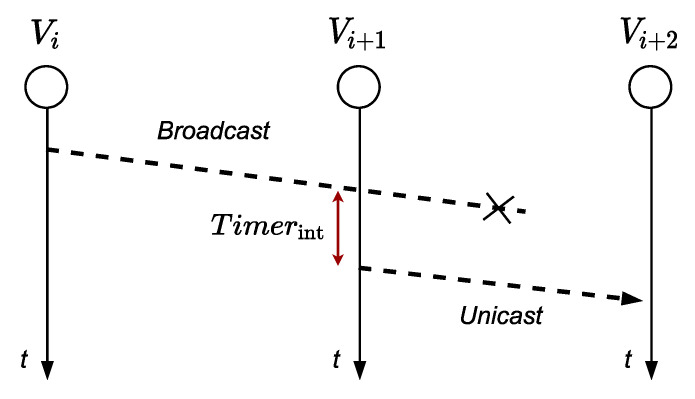
Node *v_i+_*_1_ retransmits to node *v_i+_*_2_.

The behavior of the node in noisy links will depend on whether it is a TXN, INTN, or RXN node. We present some examples that depict how our algorithm solves link problems. Consider the case where *v_i+_*_2_ does not retransmit the frame to *v_i+_*_4_ because it did not receive it or received it with errors. So, the *v_i_* or *v_i+_*_1_ node will wait a given time for one of them to retransmit the frame to *v_i+_*_2_. The node *v_i+_*_1_ will wait a time given by *timer_int_*, as seen in Figure 6, to retransmit the unicast frame to *v_i+_*_2_, storing the frame in *buffer_tx_* and placing the node address *v_i+_*_2_ in the destination direction of the frame. The node *v_i+_*_1_ retransmits first, as *timer_tx_* > *timer_int_*, to take advantage of the highest level of the signal that reaches the node *v_i+_*_2_. This retransmission will be heard by node *v_i_* concluding that the frame it previously sent did not reach *v_i+_*_2_, and it will wait until node *v_i+_*_2_ retransmits the frame. Additionally, when node *v_i+_*_2_ verifies that its address is in the destination address of the frame, it will understand that it is a retransmission made by the INTN node of the frame that TXN node sent to RXN node with broadcast address.

**Figure 7:**
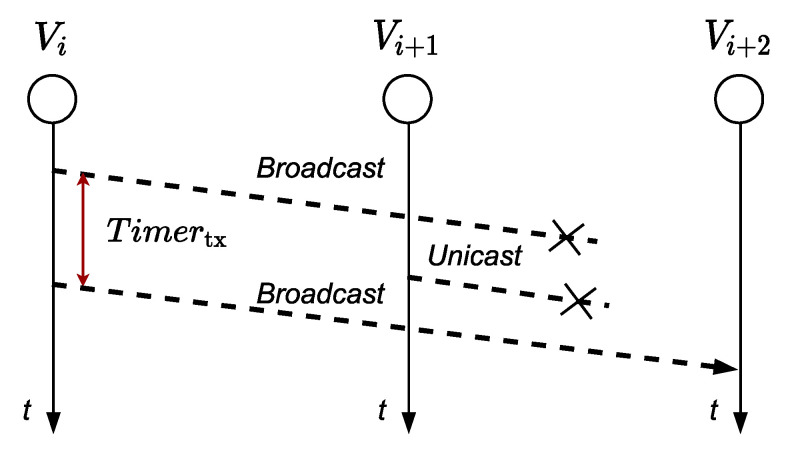
Frame does not reach node *v_i+2_*.

When the frame sent by *v_i_* does not reach *v_i+_*_2_ (Figure 7), *v_i_* starts a *timer_tx_* to retransmit the frame to *v_i+_*_2_. After this time, *v_i_* will understand that its transmission to *v_i+_*_2_ was not successful, regardless of whether the node *v_i+_*_1_ retransmitted or not the frame to *v_i+_*_2_. Therefore, node *v_i_* will retransmit the frame to *v_i+_*_2_ a maximum number of times (set by the *aMaxFrameFRameRetries* variable) using broadcast destination address in order to make information reach the nodes defined as INTN and RXN. If the maximum number of retransmissions is reached, node *v_i_* will understand that node *v_i+_*_2_ could not retransmit the frame and consequently will proceed to execute the damaged node or link procedure: changes the *FailN* variable to “TRUE” to indicate that the *v_i+_*_2_ node is failing and transmits a unicast frame to the node *v_i_*_−*1*_ indicating to act as a TXN node so it can transmit the information to node *v_i+_*_1_. In case node *v_i+_*_1_ also fails, *v_i−_*_1_ assigns the value “TRUE” to the *FailN* variable and transmits the frame to the node *v_i−_*_1_.

When the frame transmitted by *v_i_* does not reach *v_i+_*_1_ but it reaches *v_i+_*_2_, *v_i+_*_2_ retransmits the frame to *v_i+_*_4_, so this frame is also received by *v_i+_*_1_. This node discards the frame because it has determined that the flow is *Pr* and that the transmitting node is *v_i+_*_2_, concluding that the frame goes from *v_i+_*_2_ to *v_i+_*_4_ with destination node *v_n+_*_1_.

When the frame sent by node *v_i−_*_2_ reaches nodes *v_i_*, and the *FailN* variable is “TRUE”, it assumes that nodes *v_i+_*_1_ and *v_i+_*_2_ are damaged and notifies the control station using the opposite route (*P_l_*), and assigns “TRUE” to the variable *ChangeF*.

If the retransmitted frame by node *v_i+_*_2_ to node *v_i+_*_4_ does not reach *v_i_* (the situation would be similar for *v_i+_*_1_), *v_i_* waits *timer_tx_* and retransmits the frame. The receivers verify that it has already been stored in *buffer_rx_* or *buffer_ok_* and therefore they discard it. Nevertheless, node *v_i+_*_2_ will retransmit again the frame which will be discarded by the nodes *v_i+_*_1_, *v_i+_*_3_ and *v_i+_*_4_ and node *v_i_* will store it in *buffer_ok_* and then eliminate it from *buffer_tx_*.

**Figure 8:**
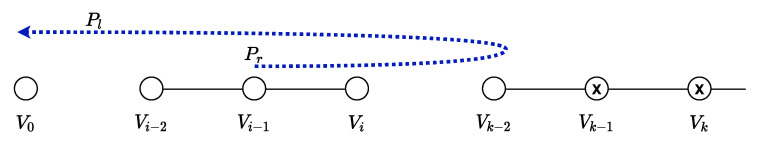
Flow *Pr* interrupted.

When a node *v_i_* sends information to the control station in the direction of the flow *Pr*, and there are at least two damaged nodes, as seen in Figure 8, the last node that works correctly (*v_k−_*_2_) will retransmit the frame in the flow towards the node *v*_0_. At that moment, when the frame passes through node *v_i_*, this node, knowing that the variable *ChangeF* is “TRUE”, will change the flow direction, starting to use *P_l_*, as the flow *Pr* is interrupted. When a frame arrives at the central station with *ChangeF* set to “TRUE”, it means that two adjacent nodes are damaged. 

**Figure 9:**
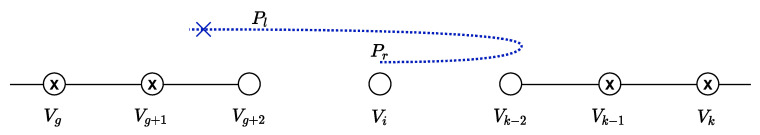
*Pr* and *Pl* flows are interrupted.

It might also happen that the node is in a network segment that has no connection with the end nodes *v_0_* and *v_n+_*_1_, because the nodes *v_g_*, *v_g+_*_1_, *v_k−_*_1_ and *v_k_* are damaged, as seen in Figure 9. Therefore, the frame would be circulating through the flows *P_r_* and finally by *P_l_* to be later discarded by *v_g_*_+2_.

When two nodes want to transmit simultaneously and the nodes are separated by two or three nodes, the problem of the hidden nodes may appear. The collision of the frames due to the problem of the hidden nodes is resolved according to what is indicated in the noisy link scenario.

### 3.3. Traffic Minimization

In linear structures, there is the possibility that several adjacent nodes detect the same event, up to five nodes that are located consecutively. Due to the value of the distance in which the nodes are separated, an event that occurred at a specific location in the linear infrastructure may cause the adjacent nodes to generate the same alarm, at the same moment in time. Therefore, they will try to notify the alarm generated to the central station, by sending a frame using the same predefined data flow, *P_r_* for example. Our proposal prevents adjacent nodes from sending repeated alarms from the same event. Before sending information related to an event, the nodes will check if that information was already transmitted by an adjacent node, confirming that the frame has already been stored in the *buffer_adj_* and avoid retransmitting it. 

### 3.4. Pseudocode and State Diagram

The Algorithm 1 that determines the operation of a node is presented below. All nodes that are part of the linear infrastructure will run the same code.
**Algorithm 1** Pseudo-code for our proposal1 **Input:**    *id_s* is source address,2 **Input:**    *id_d* is destination address, 3 **Input:**    *Maxwait*
**is** maximum wait time for an iACK from of node TXN or INTN, 4 **Input:**    *FailN* node is on 5 **Input:**    *ChangeF* The direction of traffic flow has not change6 **if** frame received **then**7          **if** frame is stored in *buffer_adj_* or *buffer_ok_* then frame is discarded8          **else**9                  **case**
*id_s* == i−1, and *id_d* == broadcast: the node is INTN, and waits iACK from node i+1:10                **case**
*id_s* == i+1: 11                **case**
*id_d* == broadcast: the node behaves as INTN and discards the frame clean *buffer_rx_* and stores frame in buffer_ok_:12                **case**
*id_d* == unicast: (i+3 node fail) node works as TXN and retransmits the frame with *id_d* = broadcast, stores the frame in *buffer_tx_*:13                **case**
*id_s* == i−2 and id*_d* == broadcast: behaves as an RXN node and retransmits the frame with *id_d* = broadcast, store the frame in *buffer_tx_*:14                **case**
*id_s* == i+2 and *id_d* == broadcast: 15                **if**
*FailN* == F **then** it has received an iACK, stores frame *buffer_ok_* and transmits the following frame **else**
16                          **case**
*ChangeF* == F, set *ChangeF* = T, transmit broadcast frame:17                          **case**
*ChangeF* == T, discard the frame:18                **end if**19          **end if**20    **else**21          **case** node INTN: retransmit unicast frame to i+1:22          **case** node TXN: 23                **if** retransmission > Maxwait. **then**
24                       transmit unicast frame to node i-1 and set *FailN* = T 25                **else** retransmit frame broadcast 26                **end if**27    **end if**

**Figure 10:**
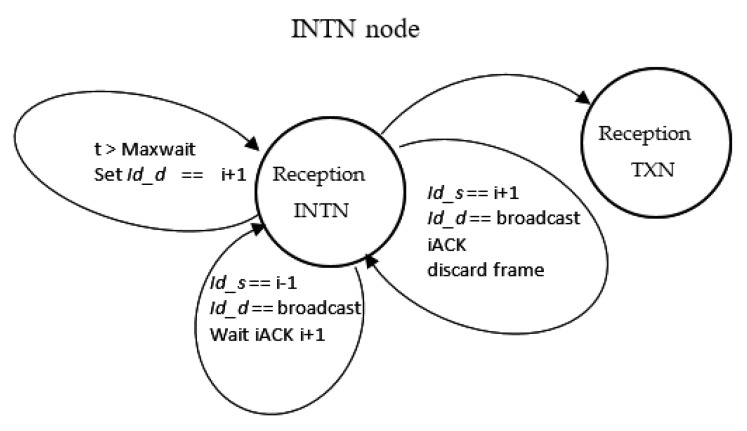
INTN node state diagram.

**Figure 11:**
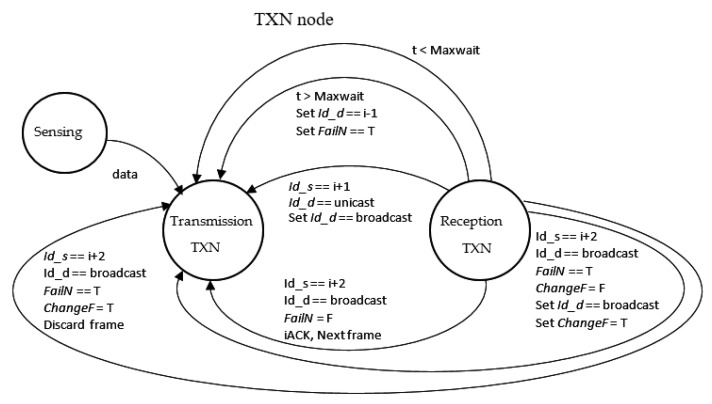
TXN node state diagram.

**Figure 12:**
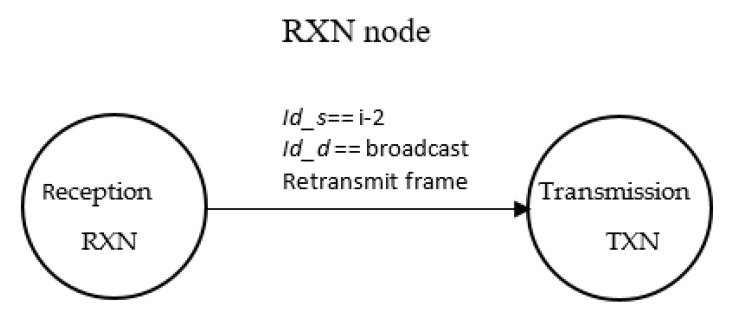
RXN node state diagram.

Figure 10, Figure 11 and Figure 12 also show the state diagram for nodes TXN, RXN, and INTN.

## 4. Energy and Transmission Time Analysis

### 4.1. System Model

To build the model, we consider a linear structure described in Section 3. For the analysis, ideal conditions are assumed: no collision, no overhead for turning on the transceiver, and prompt response in the MAC layer. Note that these assumptions are especially realistic for many LWSN networks that monitor some parameters to know the state of the infrastructure (pressure, vibrations, leaks, etc.) in vast areas: small amount of traffic, few nodes within the coverage area, environments where the presence of interference is minimal (e.g., in networks for pipe monitoring, road borders, etc.). Our proposal does not support sensors that require large bandwidths, such as cameras, radars, or lidars, that could be used to monitor especially relevant areas. Nevertheless, a separate network could be easily deployed for such traffic in the required locations. 

Although the processing of the frame for its forwarding is not negligible, we do not consider that time for the analysis because the delay caused is the same for scenarios that use iACK and eACK. Other considerations are presented below.

- All the frames have the same length, so the transmission times are fixed.
- Distance between transmitter and receiver is 50 m.
- Probability of error when transmitting a frame in each link is the same. We include a model for comparing the latency added by a retransmission using eACK or iACK. Nevertheless, for the sake of simplicity, we did not consider retransmissions in the end-to-end analysis.
- ACK frames have a constant length.
- If the transmitted data collides at the receiver, the *macAckWait* duration (an IEEE 802.15.4 attribute dependent on constants and attributes of the physical layer, which defines the maximum number of symbols to wait for an ACK frame) for the transmitting node is the sum of the turnaround time of 12 T_s_ and the ACK duration of 22 T_s_, a total of 34 T_s_ considering T_s_ = 16 μs and 1 symbol is 4 bits.
- A CCA is successful when no transmission is detected from any node, at the time of initiation of its CCA. In the IEEE 802.15.4 standard, the channel state is averaged over 8 T_s_.

### 4.2. End-to-End Transmission Time

It is important to know how much time is necessary to transmit information from a sensor to the control station. We can approximate the end-to-end delay by the sum of the time between the arrival of information at a node *v_i_* and the arrival at the *v_i+_*_2_ node [46]. In this scenario the information (alerts) is just transmitted using a single frame, and the probability that other nodes that are part of the lineal infrastructure generate alarms at the same time is very low. Therefore, this simplification will be realistic in many situations. 

Then, it is necessary to determine the time to transmit a frame when using explicit ACK, Equation (1), or implicit ACK, Equation (2):

(1) $$de\left(x\right)=T_{BO}+T_{CCA}+T_{fra}\left(x\right)+T_{TA}+T_{ACK}+T_{IFS}+\tau$$

(2) $$de\left(x\right)=T_{fra}\left(x\right)+T_{IFS}+T_{TA}+T_{BO}+T_{CCA}+\tau$$

where x represents the number of bytes that have been received from the upper layer, *T_BO_* is the back off period, $T_{CCA}$ means Clear Channel Assessment time, $T_{fra}\left(x\right)$ is Transmission time for a payload of x byte, $T_{TA}$ is Turnaround time from TX to RX, $T_{ACK}$ is Transmission time for an ACK, $T_{IFS}$ (Interframe Space time) is the Frame Processing time, *τ* is Propagation delay. 

The back off period is calculated as the product of the number of back off slots and the time for each slot (20 symbols or 320 µs). The number of back off slots is a random number uniformly in the interval (0, 2^BE^ − 1), with BE the back off exponent which has a minimum of 3. As we only assume one sender and a Bit Error Rate (BER) of zero, the BE value will not change. Hence, the number of back off slots can be represented as the mean of the interval: (2, 2^3^ − 1) or 3.5. Regarding IFS, Short IFS (SIFS) is used when the Media Access Control Protocol Data Unit (MPDU) is smaller than or equal to 18 bytes (192 µs). Otherwise, Long IFS (LIFS) is used (640 µs). Then, the transmission time of a frame with a payload of x bytes can be formulated as:

(3) $$T_{fra}\left(x\right)=8\;\left(L_{phy\;}+L_{MACHDR}\;L_{address\;\;}+x+L_{MACFTR}\right)/\;R_{data}$$

where $L_{phy\;}$ is the Length of the PHY and synchronization header in bytes (6 bytes), *L_MACHDR_* is Length of the MAC header in bytes (3 bytes), $L_{address\;\;}$ is the length of the MAC address info field, $L_{MACFTR}$ is the length of the MAC footer in bytes (2 bytes), and $R_{data}$ is the raw data rate (250 kbps). 

The transmission time for an acknowledgment can be formulated as:

(4) $$T_{ACK}=\left(L_{phy}+L_{MACHDR}+L_{MACFTR}\right)/\;R_{data}$$

So, in summary, the delay can be expressed as:

(5) de(x)=a(x)+b

In Equation (5), *a* and *b* depend on the length of the data bytes (SIFS or LIFS) and the length of the address used (64 bit, 16 bit, or no addresses). Then, the delay in the communication between a node and the edge node is a linear function of the number of bytes in the payload and the number of nodes. The MPDU is set to a maximum of 127 bytes, but the maximum number of payload bits depends on the usage of short or long addresses.

**Figure 13:**
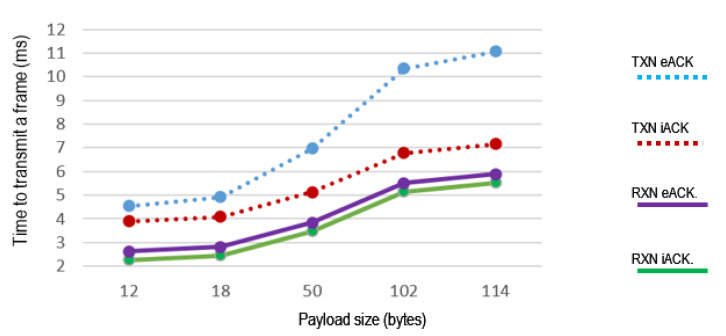
Delay according to the payload size.

Figure 13 shows the time required by the RXN node to retransmit a message, and the time required by TXN to send a second frame using eACK and iACK, B0 = 3 and short addresses scheme.

The results indicate that in the different scenarios the delay is lower when iACK is used. With a payload of 114 bytes and eACK, the delay in RXN is 5.88 ms while with iACK it is 5.53 ms. With a 12-byte payload and eACK, the delay is 2.62 ms and with iACK it is 2.27 ms. Thus, delays increase with payload length, but they are always lower with iACK. 

**Figure 14:**
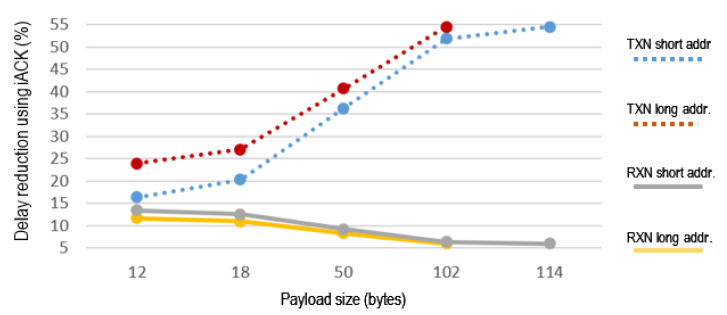
Delay reduction when using iACK compared to eACK.

Figure 14 shows how the delay decreases with the use of iACK instead of eACK, in percentages that depend on the payload, the length of the node’s address and the mode of operation of the node, TXN or RXN. The delay reduction by using iACK is higher in the TXN node. In RXN, the percentage is smaller when the payload decreases. 

When the TXN node sends a frame to the RXN node, the node expects to receive an eACK or an iACK. The node must wait a maximum time to receive these confirmations, before concluding that the sent frame did not reach its destination because of errors in the transmission. The channel error probability is independent of the acknowledgment method used, however when there are erroneous frames, the retransmission time of the frames varies according to the approach used. IEEE 802.15.4 defines the *MacAckWaitDuration* parameter, with a value of 54 Ts, as the $T_{weACK}$ time that the TXN node must wait to retransmit a frame when it does not receive an eACK.

(6) $$T_{weACK}=54\;T_{S}$$

when iACK is used, the time that the TXN node must wait to start the retransmission process of the frame, $T_{wiAck}$, is equal to the time it takes for the RXN node to finish retransmitting the entire frame plus the $T_{BO}$ and $T_{CCA}$ times. This is because the TXN node must receive the frame retransmitted by the RXN node to validate the successful transmission.

(7) $$T_{wiAck}=\;T_{BO}+T_{CCA}+\;T_{frame}$$

Even though the delay to retransmit the erroneous frame is greater with iACK, we must bear in mind that with iACK the retransmission of the frame in each node when there are no errors is 34 Ts faster, as mentioned above. As such, the time that is lost with iACK when the frame is retransmitted is compensated in the following hops. The number of hops required to make up the time lost due to iACK retransmission is shown in Table 2. Having into account that a typical linear infrastructure includes hundreds or thousands of nodes, there will not probably be any extra delay caused by using iACK instead of eACK. 

In linear structures with n nodes, the time to transmit information from node i to the extreme of the network, without collisions or lost frames in the transmission, is (for *P_l_* and *P_r_* flows):

(8) $$de_{l}\left(x\right)=\left(i\right)\;de\left(x\right)$$

(9) $$de_{r}\left(x\right)=\left(n-i\right)\;de\left(x\right)$$

Since each node knows its position or address (i) within the linear infrastructure, it will select the shortest path to reach the extreme of the network, so it will select the *P_l_* or *P_r_* flow that minimizes the delay. Table 3 shows the time to transmit from a node to the extreme of the network with a variable number of intermediate nodes. By using the iACK mechanism the time can be reduced by 9%.

### 4.3. Energy Consumption

WSN nodes are composed of several functional modules that should be modeled in order to estimate the energy consumption: microprocessor, transceiver, sensor, and power supply. The most relevant is the communication model that considers the energy consumption of the transmitter and receiver [47]. Most sensor nodes support the states *Idle*, *Transmit* and *Receive*. 

To model the energy consumed by transceiver, the average active ratio has been used. It is defined as the average active time divided by the total time spent [48]. It is a reasonable measure since energy consumption for transmission and reception (including idle listening and channel sensing) are similar in many transceivers compared to the energy consumption for standby or sleep mode [49].

An analysis has been performed to evaluate the energy consumption using iACK or eACK. Considering typical scenarios for linear structures where only alarms are transmitted, making the traffic generated very low. Let $P_{t},\;P_{r},\;P_{i}$ be the power consumed during transmission, reception and idle states respectively, and let the duration of a transmission be the sum of the duration of the data frame, ACK frame, back off, IFS, turnaround, CCA and TA. 

When using eACK, the RXN node consumes an additional amount of energy to confirm the reception of the frame.

(10) $$AE_{\mathrm{eACK}}=P_{r}T_{\mathrm{ACK}}$$

The percentage of additional energy consumed by the RXN node, AE_RXN,_ using eACK to receive the frame, transmit the ACK and be ready to retransmit the frame, can be calculated as, (Equation (11)):

(11) $$AE_{RXN}=\frac{P_{t}T_{\mathrm{ACK}}\;}{P_{r}T_{fra}+P_{t}T_{\mathrm{ACK}+}P_{i}\left(T_{TA}+T_{BO}+T_{CCA}\right)}$$

The energy consumed in the TXN node to know that the frame sent was received can be calculated for the eACK (Equation (12)) and iACK schemes (Equation (13)):

(12) $$E_{eACK}=P_{t}T_{fra}\left(x\right)+P_{r}T_{\mathrm{ACK}}+P_{i}\left(T_{IFS}+T_{TA}+T_{BO}+T_{CCA}\right)$$

(13) $$E_{\mathrm{iACK}}=P_{t}T_{fra}\left(x\right)+P_{i}\left(T_{TA}+T_{BO}+T_{CCA}\right)$$

The percentage of additional energy consumed by the TXN node, $AE_{TXN}$_,_ using eACK to send the next frame, can be calculated as Equation (14):

(14) $$AE_{TXN}=\frac{P_{r}T_{ACK}+P_{i}\left(\left(T_{waite}-T_{wait}\right)\;+2T_{TA}+T_{BO}+T_{CCA}\right)}{P_{t}T_{fra}+P_{r}T_{ACK+}P_{i}(T_{waite}\;+4T_{TA}+T_{BO0}+T_{BO1}+2T_{CCA}}$$

The percentage of additional energy consumed by the TXN node, $AE_{TXNf}$ using eACK to retransmit the frame that did not reach the RXN node, can be calculated as Equation (15):

(15) $$AE_{TXNf}=\frac{P_{i}\left(T_{waite}-T_{wait}\right)\;}{P_{t}T_{frame}+P_{i}\left(T_{waite}\;+2T_{TA}+T_{BO0}+T_{CCA}\right)}$$

Many research works have evaluated sensor node energy consumption while transmitting, receiving, or idle. In most cases energy consumption is similar in transmission or reception mode (processing and waiting) [50,51] and slightly larger than the idle mode consumption. In our experiments, and according to the specifications of our hardware [52], our node uses 55.8 mW, 49.9 mW, and 12.3 mW for $P_{t}$, $P_{r}$, and $P_{i}$ respectively, in non-beacon mode. In all cases, these values depend on the particular sensor node.

**Figure 15:**
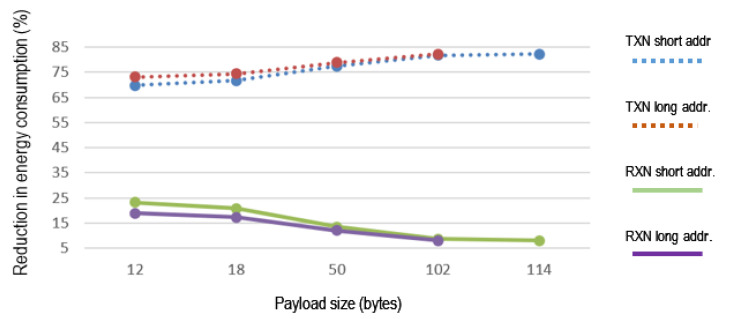
Reduction in energy consumption.

Figure 15 shows how energy consumption in the TXN and RXN nodes is reduced when iACK is used in comparison with eACK. Percentages depend on payload, node address length, and mode of operation. The percentage decrease in power consumption at the RXN node is better with small payload as opposed to the TXN node, where it is better with large payload.

Table 4 shows the percentage of energy saved when using iACK compared to the use of eACK. The reduction in energy consumption in the RXN node using iACK to retransmit the frame depends on interframe spacing. With SIFS it is about 22.5%, and with LIFS is about 8%. In TXN mode, the reduction in consumption to transmit two frames using iACK depends on the payload. With a payload of 18 bytes, it is possible to save 36% of energy, and with a payload of 114 bytes about 14%. In the RXN node the use of iACK to detect the loss of frames implies an increase of energy consumption that depends on the payload. With a payload of 18 bytes the increase is about 8% of the total energy consumed by the node and with a payload of 114 bytes the increase is about 2%.

Thus, by confirming the reception of the information using iACKs we can reduce the consumption of energy [28]. When the transmission reliability is left for superior layers, where the *v_n+_*_1_ node confirms the message reception to the node *v_i_*, the energy consumption affects all the intermediate nodes that transmit the ACK confirmation.

## 5. Implementation

**Figure 16:**
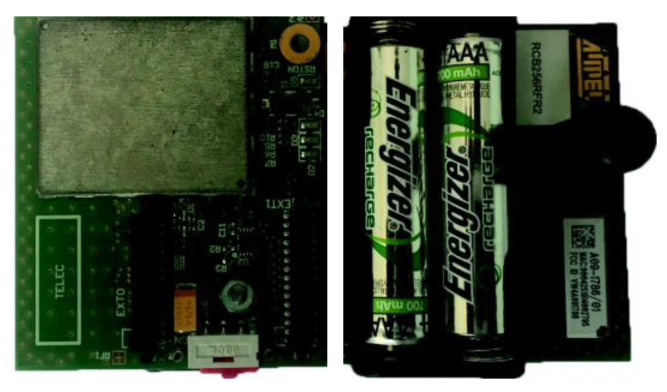
Atmel RCB256RFR2 node.

We have performed a practical validation of the proposed architecture using an Atmel RCB256RFR2 device [53], which includes a compatible IEEE 802.15.4 transceiver (Figure 16).

The MAC layer was implemented with Atmel Software Framework (ASF) [54], which simplifies the control of the contents of the payload and the header of the frame.

**Figure 17:**
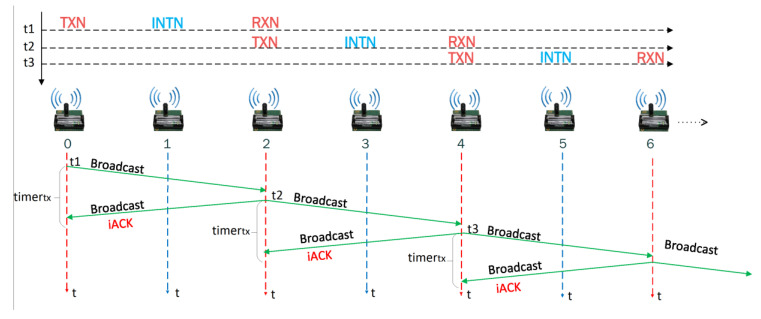
Tested network.

In our test, nodes operate as Full Function Devices (FFDs), and are arranged in a linear topology with seven nodes, as shown in Figure 17.

**Figure 18:**
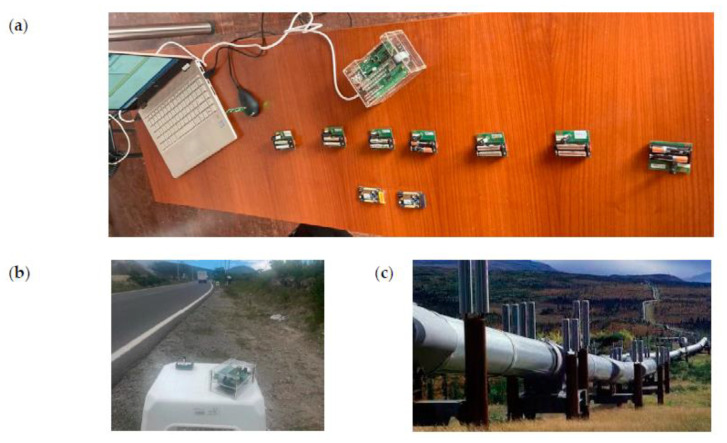
(**a**) Used hardware. (**b**) Scenario for the tests. (**c**) Typical pipeline implementation.

Figure 18a shows the hardware used in the experiments (Atmel RCB256RFR2 nodes). One of the environments where the measurements were completed is shown in Figure 18b). Previously, we validated the algorithm in the laboratory with three nodes working as TXN, RXN, and INTN, in a controlled environment, where sniffers were used to analyze the traffic among the nodes. Then, we used 7 nodes to build a larger structure. Although the expected communication range with IEEE 802.15.4 would be approximately 70 m, we placed each node 50 m (line of sight) from each other to ensure connectivity. The obtained measurements can be used to estimate the results in an infrastructure of hundreds of nodes, like the required for a scenario such as the shown in Figure 18c).

Table 5 shows the power levels and duration of the frames used in different the tests. A sniffer was used to capture IEEE 802.15.4 frames in order to check the correct operation of the algorithm to transmit information to the control center, even when there are retransmissions or interruptions due to damaged nodes, noisy links, or links that work intermittently. Such scenarios are forced by modifying the code of some nodes.

Failures in the link are simulated by forcing the nodes to not transmit the information when they should (according to the algorithm), and some nodes are disconnected to simulate a node that failed completely.

**Figure 19:**
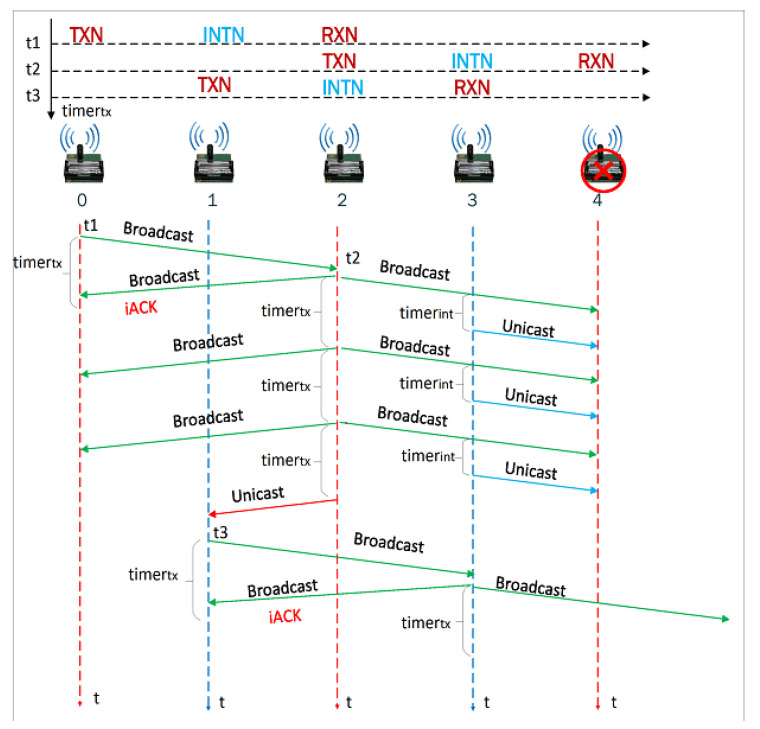
Test with a failing node.

Different tests were completed to check the correct operation of the algorithm in those scenarios. For example, Figure 19 shows a test where the connectivity recovery is performed when a node is damaged. In this scenario, node *v_o_* transmits data to node *v*_2_, using a broadcast frame, node *v*_2_ retransmits it to node *v*_4_, since node *v*_4_ is broken, node *v*_2_ does not receive an iACK and therefore retransmits the frame three times.

Node *v_o_* received the iACK from node *v*_2_ so it does not retransmit the information. After the third attempt, node *v*_2_ transmits a unicast frame to node *v*_1_ indicating that node *v*_4_ is damaged. Therefore, node *v*_1_, which was operating as an INT node, will operate as a TXN node and retransmit the frame to node *v*_3_ that, in turn, transmits to node *v*_5_, solving the problem of the failed node *v*_4_. In case the node *v*_3_ is also broken, node *v*_1_ will discover that nodes *v*_3_ and *v*_4_ are failing and it will change the direction of the flow of the transmission.

In our approach, the delay for a frame in the node, with a payload of 12 bytes is 2.43 ms (without considering the frame processing time), the average time in the RXN node to retransmit a frame, operating with iACK is 3.4 ms. This value is similar to the average retransmission time in a TelosB node running TinyOS, which is 3.63 ms [55].

To measure the frame processing time, we used a RCB256RFR2 node, which includes an IEEE 802.15.4 transceiver. The MAC layer was implemented using Atmel Software Framework (ASF), which provides direct access to the payload and the header of the frame, without requiring an operating system in the node. This feature allowed us to measure delays using iACK and eACK with IDnode = IDPAN = 2 bytes, BO = 3 and with a frame processing time equal to LIFS 0.640 microseconds. Table 6 and Table 7 show how there is also a small reduction in the processing time when using iACK both in the node working as TXN or the node working as RXN. This also helps saving energy. The difference in the frame processing time is related to the low-level operation of the software stack of the node.

Other protocols, such as AODV, HTR [56] COLBA, ABORT [57], RPL, or 6LowPAN [58] require higher times. For example, we can compare our proposal with 6LowPAN, a protocol that also provides address autoconfiguration, is robust against link failure or device unavailability, and that is designed for large multi-hop networks. Table 8 shows the end-to-end delay measured in [58] using 6LowPAN and using our proposal. 6LowPAN is a protocol designed for multi-hop networks, but it is not specifically adapted to large-scale multi-hop linear topologies, causing high delays because of retransmissions in higher transport layers.

It is also possible to place nodes closer than the maximum communication range to reduce the transmission power and reduce the probability to require retransmissions. For example, nodes can be placed in each one of the segments that are used to build the pipeline. Typically, those segments have a maximum length of 15 m.

## 6. Conclusions

This paper presents a new proposal for sensing linear infrastructures with WSN to send alerts. By taking advantage of the physical topology imposed by the infrastructure, we use implicit ACKs (iACKs) to reduce energy consumption and the time to transmit information to the edge of the linear segment (connected to the control center of the infrastructure). The routing also takes advantage of the particularities of the linear topology. We described the operation of the algorithm in a typical scenario and in the presence of problems such as noisy links or with inoperative nodes, showing how the algorithm can solve the problem to forward the information to the edge of the network.

After studying different existing proposals, we observe that our algorithm covers almost all the functions typically related to the network layer. Therefore, by using our algorithm at the link level layer allows for eliminating the network layer in a linear topology, leaving the few functions that are not performed, such as flow control, to the transport or application layer.

We have modeled the time required to transmit alerts from a node to the control center in order to check if this is a suitable algorithm for large infrastructures. The results show that our algorithm requires approximately 1 s to cross a 10 Km section, and that by using iACKs we can reduce the time by a 9% compared to using eACKs. Furthermore, the use of iACKs also decreases the energy consumption.

Finally, we have implemented our algorithm using Atmel RCB256RFR2 nodes to test its operation in a real scenario. We have completed different tests that included noisy links and failing nodes. We could check how our proposal provides reliable transmission and low latency for linear sensor networks, supporting failing nodes or noisy links, while simplifying the network layer. Thus, this algorithm is especially interesting for implementing pipeline monitoring applications over a 802.15.4 physical layer.

## Figures and Tables

**Table 1:** Comparison of LPWAN protocols.

	Address Autoconfig.	Same Net. and Link Addr.	eACK	iACK	Linear Topology	Clustering	End-to-End Reliability	Topology Discovery	Energy Efficient	Considers End-to-End Delay	Large Scale	Access Channel	Protection against Link and Node Failure
This proposal	yes	yes	no	yes	yes	no	yes	yes	Yes	yes	yes	CSMA/CA	yes
			Application and transport level										
RCRT [23]	no	no	yes	no	no	No	yes	no	No	yes	no	n.a	yes
GARUDA [25]	no	no	yes	no	no	Yes	yes	no	n.a.	no	yes	n.a	yes
ATP [26]	no	no	yes	no	no	No	yes	no	No	yes	yes	n.a	no
SCTPWSN [24]	no	no	yes	no	no	No	yes	yes	n.a.	no	yes	n.a.	yes
					Network level								
SimpliMote [34]	no	yes	no	no	yes	No	no	yes	yes	no	yes	n.a.	yes
MERR [35]	no	no	no	no	yes	No	no	no	yes	no	yes	n.a.	no
LDG [36]	no	no	no	no	no	Yes	no	yes	yes	yes	yes	n.a	no
DiscoProto [10]	yes	yes	no	no	no	Yes	no	yes	no	yes	yes	TMAC	yes
ACO&GA [37]	no	no	no	no	yes	No	no	no	yes	no	yes	n.a.	no
LBHRP [2]	no	no	no	no	no	Yes	no	yes	no	no	yes	TDMA	yes
ROLS [6]	yes	no	no	yes	yes	Yes	no	no	no	no	yes	n.a.	yes
WTDP [29]	yes	no	no	no	no	No	no	yes	no	no	no	aloha	no
					Link level								
PRIMAC [38]	no	no	yes	no	yes	No	no	yes	no	no	yes	n.a	no
SA-MAC [39]	no	no	yes	no	yes	Yes	yes	yes	no	yes	no	TDMA	no
RP MAC [40]	no	no	yes	no	yes	No	no	no	yes	yes	no	n.a.	no
HEPRMAC [41]	no	no	no	no	no	No	no	no	yes	yes	no	TDMA	no
Token [42]	no	no	yes	no	yes	Yes	no	no	n.a.	yes	no	Token	no
LCMAC [13]	no	no	yes	no	no	No	yes	no	yes	yes	yes	n.a.	no
MIMAC [16]	no	no	yes	no	yes	No	no	no	yes	yes	yes	CSMA/CA	no

**Table 2:** Number of hops required to balance out a retransmission with iACK compared to eACK according to the payload size.

Payload	Required Hops
12	1
18	1
50	3
102	6
114	7

**Table 3:** Time for end-to-end transmission with 16 bits ID.

Intermediate Nodes	LWSN Length (Km)	eACK Delay (s)	iACK Delay (s)
100	3	0.33441	0.30721
1000	30	3.3441	3.0721
2000	60	6.6882	6.1442
3000	90	10.0323	9.2163
4000	120	13.3764	12.2884

**Table 4:** Variation of energy consumption in the node with ID = 16 bits

Payload	$AE_{RXN}$	$AE_{TXN}$	$AE_{TXNf}$
18	22.5%	36%	−8%
114	8%	14%	−2%

**Table 5:** Design parameters.

Parameter	Description
Transmission power	0.5 dBm
Distance between nodes	25 m
802.15.4 broadcast frame TXN	0.384 ms, 12 bytes

**Table 6:** Frame processing time (RXN node).

	Processing Time at Node RXN		
	Measured [ms]	Calculated [ms]	% error
eACK	3.78	3.32	12%
iACK	3.43	3.27	4.6%
Difference	0.35	0.05	

**Table 7:** Frame processing time (TXN node).

	Processing Time at Node TXN		
	Measured [ms]	Calculated [ms]	% error
eACK	6.98	6.33	9.31%
iACK	6.66	5.82	12%
Difference	0.32	0.51	

**Table 8:** Comparison of the end-to-end delay between 6LowPAN and our proposal.

	End to End Delay		
Hop	Our proposal	6LowPAN	LWSN length
1	3.43 ms	13.88 ms	50 m
10	34.3 ms	138.8 ms	275 m
100	343 ms	1.38 s	2.525 km
1000	3.43 s	13.88 s	25 km
2800	9.06 s	38.86 s	70 km

## Data Availability

The source code used for the practical validation of the proposed architecture is available at https://github.com/cegas/routinglinklevel.
